# Effect of romosozumab in premenopausal women with severe osteoporosis and anorexia nervosa

**DOI:** 10.1016/j.afos.2023.10.001

**Published:** 2023-11-18

**Authors:** Kazuki Fujimoto, Narumi Maki, Daisuke Hashiba, Toshifumi Maeyama, Ryosuke Nakagawa, Hajime Arai, Seiji Ohtori

**Affiliations:** a Department of Orthopaedic Surgery, Kohnodai Hospital, National Center for Global Health and Medicine, 1-7-1 Kohnodai, Ichikawa City, Chiba, 272-8516, Japan; b Department of Orthopaedic Surgery, Graduate School of Medicine, Chiba University, 1-8-1 Inohana, Chuo-ku, Chiba, 260-8670, Japan

**Keywords:** Anorexia nervosa, Secondary osteoporosis, Anti-sclerostin antibody, Bone metabolism

## Abstract

**Objectives:**

This study aims to investigate the effects of romosozumab on bone mineral density (BMD) and bone metabolism.

**Methods:**

In this retrospective case series, romosozumab was administered to 5 premenopausal female patients with osteoporosis and anorexia nervosa with fragility fractures. BMD and bone turnover marker changes were investigated at 6 months and 1 year after administering romosozumab.

**Results:**

BMD increased and high-turnover bone metabolism decreased 6 months and 1 year after administering romosozumab.

**Conclusions:**

Romosozumab is useful for treating osteoporosis in patients with anorexia nervosa.

## 1 Introduction

Women with anorexia nervosa (AN) have been reported to develop osteoporosis. Diet therapy for weight gain and resumption of menstruation is effective in treating osteoporosis, but only 50%–60% of patients recover their bone mineral density (BMD) to a sufficient level [1,2]; therefore, drug therapy should be considered. Bisphosphonates and teriparatide have been found to increase BMD in women with osteoporosis and AN [[3], [4], [5]]; however, the effect of *anti*-sclerostin antibodies is unknown. This study aims to investigate the effects of romosozumab on BMD and bone metabolism. In the current study, we administered romosozumab to premenopausal women with osteoporosis and AN, and investigated its effects on BMD and bone metabolism.

## 2 Methods

This retrospective case series study was conducted as part of the “Research of muscle mass loss in patients with osteoporotic fractures,” an observational study of BMD and muscle mass in patients with osteoporotic fragility fractures. This study was approved by our facility's Institutional Review Board for Clinical Research (NCGM-S-004297-00), and informed consent was obtained from all study participants.

### 2.1 *Participants*

From April 2019 to June 2022, romosozumab was administered to 5 women aged 31–41 years with osteoporosis and fragility fractures undergoing psychosomatic treatment for AN and followed up for at least 1 year. According to the diagnostic criteria of the Japanese Society for Bone and Mineral Research and the Japan Osteoporosis Society, romosozumab is indicated for patients at high risk of fracture, and premenopausal women are not excluded. Therefore, no specific consent was given to patients who met the diagnostic criteria of “bone mineral density ≤ −2.5 SD and at least 1 fragility fracture”.

The following additional inclusion and exclusion criteria were applied:

Inclusion criteria.

1. Patients who met the diagnostic criteria for primary osteoporosis according to the guidelines of the Japan Osteoporosis Society.
2. Those with BMD (of either the lumbar spine or femoral neck) 2.5 standard deviations or less from the mean (T-score of −2.5 or less) or 2.0 standard deviations or less from the standard for the same age group (Z-scores of −2.0 or less), indicating osteoporosis.

Exclusion criteria.

1. Patients with diseases that affect gonadal hormones.
2. Those on steroid treatment.
3. Those with malignant tumors.

### 2.2 *Study setting: bone densitometry was performed at our facility*

Research procedures and equipment: Dual-energy X-ray absorptiometry (Discovery: Hologic, Waltham, MA, USA) was performed to measure the lumbar spine and femoral neck BMDs before and 6 months and 1 year after administering romosozumab. Biochemical markers of bone turnover, such as tartrate-resistant acid phosphatase-5b (TRACP-5b) and procollagen type 1 N-terminal propeptide (P1NP), were measured before and 6 months and 1 year after administering romosozumab.

### 2.3 *Evaluation items*

1. Patient characteristics: Age, sex, height, weight, body mass index (BMI), serum 25-hydroxyvitamin D (25-OHD), previous fracture site, years since the onset of AN, and previous use of bisphosphonates, resumption of menstruation
2. Changes in body weight and skeletal muscle mass index (SMI; limb muscle mass (kg)/height (m)^2^)—an efficient parameter to exclude the effects of height, scrutiny, and racial differences in limb muscle mass [6]—before and 6 months and 1 year after administering romosozumab.
3. The presence or absence of sarcopenia was diagnosed using the Sanada classification of SMI, with a cut-off value of 5.4 kg/m^2^ for women [7].
4. Primary endpoint: BMD changes at 6 months and 1 year after administering romosozumab
5. Secondary endpoint: Changes in bone turnover marker levels at 6 months and 1 year after administering romosozumab (normal values for premenopausal women [30–44 years]: TRACP-5b, 120–420 mU/dL; P1NP, 16.8–70.1 ng/mL)

### 2.4 *Statistical analysis*

The paired *t* test was used to determine the significance of temporal changes in BMD and bone turnover marker levels. P-values < 0.05 were considered statistically significant.

## 3 Results

### 3.1 *Patient characteristics before administering romosozumab*

**Fig. 1:**
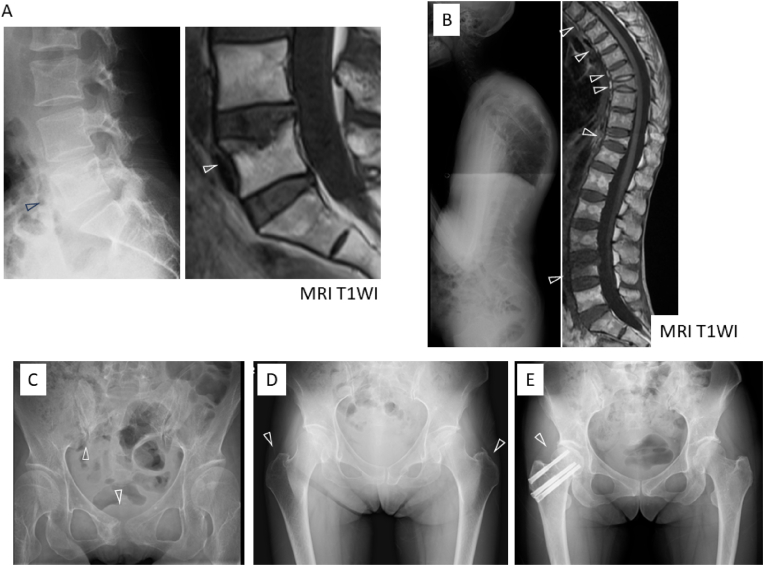
The images of past fractures. X-rays and T1 weighted images (T1WI) of magnetic resonance imaging (MRI) A: case 1, An L5 compression fracture. B: case 2, Multiple thoracolumbar compression fractures (T3, 5, 7, 8, 11, L4). C: case 3, Right pubic and sacral fractures. D: case 4, Bilateral femoral greater trochanteric fractures. E: case 5, A right femoral neck fracture.

Table 1a shows the patient characteristics before administering romosozumab. The mean age of the patients was 37.3 ± 4.3 years. The average BMI was 14.0 ± 3.2 kg/m^2^, indicating a low body weight in all patients. The average number of years since the onset of AN was 15.0 ± 6.4 years. The average 25-OHD level was 19.7 ± 10.6 ng/mL; 2 patients showed 25-OHD levels of 8.4 ng/mL and 8.5 ng/mL, respectively, indicating vitamin D deficiency. All patients had sarcopenia, assessed using the SMI during treatment. Body weight did not change significantly during the treatment (Table 1b). X-ray images of past fractures in all 5 cases are shown in Fig. 1.

**Table 1A:** Clinical characteristics of the patients at pretreatment.

Patient number	Age (yr)	Sex	Height (cm)	Weight (kg)	BMI (kg/m^2^)	Serum 25OHD (ng/ml)	Past fracture site	Years since the onset of AN	Past use of Bisphosphonate	Resumption of menstruation
1	41	F	150.2	22.8	10.1	8.4	Vertebral body (L5)	10	8 months	–
2	31	F	148.4	27.8	12.6	23.0	Vertebral body (T3, 5, 7, 8, 11, L4)	16	None	–
3	41	F	160.0	32.0	12.5	28.2	Pubis and sacrum	21	None	–
4	36	F	156.0	41.0	16.8	30.4	Femoral greater trochanter	7	7 years	+
5	40	F	163.0	47.0	17.7	8.5	Femoral neck	21	None	+
Mean	37.8		155.2	34.1	14.0	19.7		15.0		
SD	4.3		6.2	9.8	3.2	10.6		6.4		

SD, standard deviation; BMI, body mass index, 25OHD, 25 hydroxyvitamin D, AN, anorexia nervosa.

**Table 1B:** Body weight and SMI change after treatment.

Patient number	Body weight (kg)			SMI (kg/m^2^)		
	Before administering romosozumab	6 months later	1 year later	Before administering romosozumab	6 months later	1 year later
1	22.8	29.4	23.0	3.3	3.8	3.1
2	27.8	27.8	34.0	5.5	4.5	4.8
3	32.0	28.6	28.0	3.9	3.5	3.4
4	41.0	43.0	44.0	–	4.9	5.1
5	47.0	47.0	47.0	4.8	4.8	4.9
Mean ± SD	34.1 ± 9.8	35.2 ± 9.1	35.2 ± 10.2	4.4 ± 1.0	4.3 ± 0.6	4.3 ± 0.9
P-value		0.56	0.56			

SD, standard deviation; SMI, skeletal muscle mass index.

### 3.2 *Primary endpoint (BMD changes before and 6 months and 1 year after administering romosozumab)*

Before administering romosozumab, the BMDs of the lumbar spine and femoral neck were 0.593 ± 0.193 g/cm^2^ and 0.403 ± 0.075 g/cm^2^, respectively, whereas, at 6 months, they were 0.679 ± 0.172 g/cm^2^ (P = 0.01) and 0.420 ± 0.095 g/cm^2^ (P = 0.60), respectively, showing a significant increase at the lumbar spine. Similarly, the BMDs of the lumbar spine and femoral neck increased at 1 year after administration of romosozumab (lumbar spine, 0.713 ± 0.180 g/cm^2^, P = 0.00; femoral neck, 0.451 ± 0.075 g/cm^2^*,* P = 0.13) compared to those before administration. Although the difference was not significant for the femoral neck, the BMDs increased by 15% and 5% at 6 months and 20% and 12% at 1 year after administering romosozumab in the lumbar spine and femoral neck, respectively. Despite the lack of significant changes in body weight or SMI, a significant increase in the BMD of the lumbar spine was observed after romosozumab administration (Table 2) (see Table 3).

**Table 2:** BMD changes after treatment.

Patient number	Lumbar BMD (g/cm^2^)			Femoral neck BMD (g/cm^2^)		
	Before administering romosozumab	6 months later	1 year later	Before administering romosozumab	6 months later	1 year later
1	0.478	0.628	0.668	0.339	0.331	0.421
2	0.438	0.520	0.524	0.417	0.547	0.513
3	0.491	0.575	0.620	0.314	0.327	0.366
4	0.653	0.714	0.756	0.456	0.420	0.409
5	0.906	0.959	0.999	0.490	0.475	0.547
Mean ± SD	0.593 ± 0.193	0.679 ± 0.172	0.713 ± 0.180	0.403 ± 0.075	0.420 ± 0.095	0.451 ± 0.075
P-value		0.01*	0.00*		0.60	0.13

*P-value <0.05; statistically significant compared to before administering romosozumab.

SD, standard deviation; BMD, bone mineral density.

**Table 3:** Biochemical markers of bone turnover change after treatment.

Patient No.	Serum TRACP-5b (mU/dL)			Serum P1NP (ng/ml)		
	Pre	6 months later	1 year later	Pre	6 months later	1 year later
1	641	187	72	74.4	49.5	23.4
2	394	–	159	186	–	25.5
3	539	295	173	150	59.2	30.3
4	169	147	77	21.4	51.8	16.1
5	104	–	94	28.1	–	36.2
Mean ± SD	369.4 ± 231.2	209.7 ± 76.6	115.0 ± 47.5	92.0 ± 73.4	53.5 ± 5.1	26.3 ± 7.5
P-value			0.06			0.11

*P-value <0.05; statistically significant compared to before administering romosozumab.

SD, standard deviation; TRACP-5b, tartrate-resistant acid phosphatase-5b; P1NP, procollagen type 1 N-terminal propeptide.

### 3.3 *Secondary endpoint (changes in bone turnover markers before and 6 months and 1 year after administering romosozumab)*

The bone metabolism marker levels before and 6 months and 1 year after administering romosozumab, respectively, were as follows: TRACP-5b: 369.4 ± 231.2, 209.7 ± 76.6, and 115.0 ± 47.5 mU/dL; P1NP: 92.0 ± 73.4, 53.5 ± 5.1, and 26.3 ± 7.5 ng/mL. The levels of high-turnover bone resorption and formation markers decreased following romosozumab administration; however, no significant difference was noted.

## 4 Discussion

This study revealed two findings: romosozumab administration increased the BMD and improved the high-turnover bone metabolism marker levels in premenopausal women with severe osteoporosis and AN who presented with fragility fractures.

### 4.1 *Romosozumab administration significantly increased the BMD of the lumbar spine in premenopausal women with severe osteoporosis and AN*

Previous studies have shown that 38% of patients with AN have osteoporosis and a 3-fold risk of fracture [8,9] and 57% of women with AN experience at least 1 fracture in their lifetime [10]. In patients with AN with amenorrhea as a complication, 2.6% and 2.4% of the bone loss occurs in the spine and femur, respectively [11], and ensuring weight gain and resumption of menstruation is the main treatment strategy. However, only 50%–60% of patients recover sufficiently [1,2]; therefore, additional treatments, such as drug therapy, should be considered. Oral estrogen does not restore BMD in adult women [12,13]. Bisphosphonate administration causes a 3%–4% increase in the lumbar spinal BMD and a 2% increase in the femoral BMD; however, long-term administration should be avoided, especially in young patients [3,4]. Teriparatide leads to 6%–10% increase in the spinal bone mass [5].

The present study revealed that despite there being no significant increase in body weight or SMI, the BMDs of the lumbar spine were significantly increased at 6 months and 1 year after administering romosozumab. Thus, romosozumab was deemed useful for the treatment of osteoporosis in our patients with AN.

### 4.2 *Romosozumab administration improved the high-turnover bone metabolism markers in premenopausal women with severe osteoporosis and AN*

It has been reported that patients with AN have high sclerostin levels, decreased bone formation marker levels, and increased bone resorption marker levels [[14], [15], [16], [17], [18]]; however, the detailed mechanism remains unclear.

In the present study, the levels of bone formation and resorption markers were increased, indicating a high turnover of bone metabolism in all patients, except in 2 in whom menstruation had resumed. Romosozumab led to reduced bone resorption and bone formation marker levels at 6 months and 1 year after administration.

It has been reported that after romosozumab administration, bone formation rapidly increases and returns to baseline by 9 months, and bone resorption decreases earlier and remains low [19,20]. The decrease in bone formation at 6 months after administering romosozumab indicated recovery after a rapid increase.

## 5 Conclusions

The BMD of the lumbar spine significantly increased at 6 months and 1 year after administering romosozumab in premenopausal women with severe osteoporosis and AN who presented with fragility fractures. Romosozumab improved the levels of high-turnover bone metabolism markers. Romosozumab can be an effective treatment for osteoporosis in premenopausal underweight women, not in only those with AN but also in those with other diseases that cause underweight. However, these findings were based on data from a small number of patients; therefore, accumulating data from a large number of patients is necessary.

## CRediT author statement

**Kazuki Fujimoto:** Conceptualization, Methodology, Writing – original draft. **Narumi Maki:** Investigation, Writing – review & editing. **Daisuke Hashiba:** Investigation, Writing – review & editing. **Toshifumi Maeyama:** Data curation, Writing – review & editing. **Ryosuke Nakagawa:** Writing – review & editing. **Hajime Arai:** Supervision. **Seiji Ohtori:** Supervision.

## Conflicts of interest

The authors declare no competing interests.
